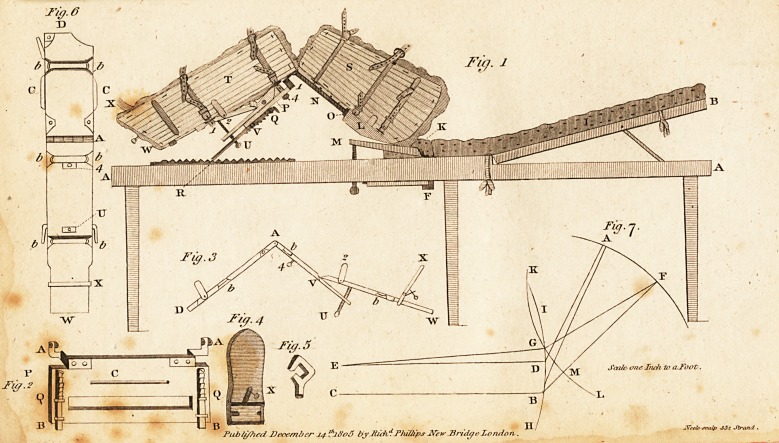# Observations on Select Subjects in Surgery

**Published:** 1806-01

**Authors:** 


THE
Medical and Phyfical Journal.

VOL. XV.] JANUARY, 1806. [NO. 83.

*Printed for R. PHILLIPS, by W. Thorne, Red Lion Court, Fleet Street, London.*

### OBSERVATIONS ON SELECT SUBJECTS IN SURGERY.

BY MR. SIMMONS.

( Continued from Vol. xiv. pp. 481—490. )

### ON A CASE REQUIRING THE USE OF THE CROTCHET.

A Given disproportion between the head of the child and the diameter of the pelvis of the mother, constitutes a legitimate cause for the use of the crotchet. This disproportion may arise from the incompressibility or preternatural enlargement of the head itself, or from a malformation or morbid contraction in the passage of the pelvis. Either of these instances singly, and especially the union of two of them in the same subject, may render it expedient to open the head, in order to reduce its bulk, and restore the equilibrium between the body to be extruded and the cavity through which it has to pass. Uncontested as are these points of doctrine, their right application to the case before us is sometimes very difficult. In every such instance, the operator will be relieved from much painful anxiety by the death of the child; and the body, loosened in its texture by putrefaction, will oppose less resistance to its subsequent delivery. Yet, where the obstruction is still greater than is here supposed, and the labour consequently more lingering, the long continued pressure of the head may induce a mortification of the uterus, or soft parts lining the pelvis. To the mother this is a source of danger that has met with less attention from practitioners than, in my opinion, it is entitled to receive. The early indications of it are those of inflammation, which is sometimes, though not always, ushered in by pyrexia, the pulse becomes hard and frequent; the pains almost incessant, are described to be like the cutting of a sharp instrument; the exclamations too are loud and shrill, expressive of distress, and accompanied with an effort to draw up rather than bear down during a pain, as it is usual in the several stages even of a hard labour. On the occurrence of these symptoms, it will be proper to determine on the early removal of the occasional cause, or delay may prove fatal to the mother, as the following case will exemplify.

A middle-aged woman, who had born a living child at her first birth, and had afterwards been delivered by the crotchet, was again taken in labour at the full time.—From the beginning, she was under the care of an intelligent and experienced midwife, at whose request I saw her, because for many hours the head had remained immovably fixed at the superior aperture of the pelvis, on the right side, notwithstanding the pains had been regular and strong. Externally she exhibited appearances of distortion; her legs were curved anteriorly; and such was the narrowness of the pelvis, as to afford no rational hope of the birth of a full grown living child. At the time of my first visit the child was probably alive, therefore it was proposed to wait some time longer, lest the strong measures likely to become necessary should bear the appearance of precipitation. But on the following day she was seized with a rigour, the pulse became frequent and hard, the pains strong and almost continual, and so cutting as to produce the most piercing exclamations. In this extremity she was copiously bled from the arm,* which afforded her considerable relief; and glysters, anodynes, and other suitable remedies were enjoined. From the woman's own testimony, and other evidence, it could hardly be doubted that the child had now been dead several hours; and as the head still retained its full rotundity, I proposed to open it: but in this I was over-ruled by the decision of an experienced surgeon, who had seen her at intervals during the labour; because, in his opinion, as the midwife informed me, the death of the child would diminish the resistance, and supersede the use of the crotchet. Agreeably to this prediction, I was sent for in haste, thirty-six hours afterwards, and just in time to receive the body, which was expelled with so much force as to separate the funis from the placenta. After waiting in vain for the descent

* In a case of topical inflammation the coagulum has an opake white crust upon its surface, not unlike the rind on bacon; while in blood drawn during pregnancy it is of a bluish tint, and less cohesive.

scent of the placenta, I introduced my hand, and found it detained in the upper chamber of the uterus by the hour glass contraction; to overcome which, gentle efforts were sufficient, and the whole was brought away, likewise putrid. All this she bore with surprising fortitude; but soon after the pulse became very quick and feeble, the respiration high and laborious, and in about an hour and a half after her delivery she expired.

I opened the body in the presence of two medical gentlemen. A mortification had taken place in the uterus, both anteriorly and posteriorly, opposite the points where the head of the child had pressed it against the bones of the pelvis, at the superior aperture.

Here the unyielding resistance of the head had obviously proved the cause of death to the mother. For the most part, excepting the pelvis is wide, or the head relatively small, the bones of the fetal cranium lap over each other in the birth, and are moulded into a conoidical form. If the ossification should be too complete to admit of this alteration of figure, much difficulty may be expected; and if the pelvis is also much narrowed hy distortion, still more serious apprehensions may be justly entertained. In the latter case, a wish to save the life of the child can alone prevent an early recurrence to the crotchet. But, when the child is known to be dead, surely the resting of the head upon the inflamed uterus, even for a few hours longer, while putrefaction is taking place, as inductive to the most fatal consequences, ought not to be allowed.

The danger to the mother from inflammation will be augmented in proportion to the increased resistance of the living head, without the diminution of which there can be no chance of relief to her. When, however, in this extreme case, the safety of the mother demands the employment of the crotchet, let it not be used without the most deliberate consideration, and then only on the fullest conviction of its propriety and necessity.

### On a Luxation of the Olecranon.

Among the accidents to which the joint of the elbow is exposed, a luxation of the olecranon may be enumerated; I have met with only a solitary instance of it.

A boy, ten years of age, fell from the back of his playfellow upon the elbow of his left arm: upon the supposition of its being dislocated, extension was made in the customary manner without success, and I was requested to see him. Instead of a complete luxation of the joint, it appeared to me that the olecranon only had been displaced, in effecting which the interosseous ligament had been lacerated. Therefore, after placing the limb in a state of semi-flexion, with a very moderate degree of force applied on its outer surface, I reduced the olecranon into its original position.

### On the Division of the Iris.

It is a maxim in surgery to use as few instruments as possible, as well as to prefer those of the most simple structure. The iris is sometimes so much contracted as to obstruct or destroy vision; and it has then been recommended to divide it with a small knife, or even to cut out a small segment of it with a pair of small scissars; but where the disposition to contraction is still strong, the opening thus made may again close, and frustrate the benefit to be expected from the operation. In a recent case in which this operation was thought adviseable, I used the couching needle of Mr. Hey.

A young man, who had lost the left eye by the small-pox, had also a large opacity over the transparent cornea on the right, almost covering the pupil, which was likewise contracted to the size of a small pin's head, and immoveable. He could see a little, and it was thought that his range of vision would be extended by enlarging the aperture. With this view I pushed the needle into the eye behind the iris, with the flat side towards it, and passing the point through the pupil, I carried it forward anteriorly to the circumference of the iris next the inner canthus, when, turning the edges of the instrument horizontally, the iris on that side was completely divided by the inner edge, and with a sound distinctly audible. Owing to a distortion in the eye itself, next the outer canthus, a very narrow border only of the iris was left; this I endeavoured to catch upon the point of the instrument, but it was now so loose as to endanger wounding the transparent cornea near the centre of it in making the attempt; I therefore desisted, proposing, if hereafter it should be thought expedient, to divide it directly from the outer canthus, and by introducing the instrument anteriorly to the iris. But little inflammation ensued; I fear however, that there is a cataract behind, for which a further operation may become necessary.

On

### On the Phlegmasia Alba.

The disease which has passed under the title of œdema lacteum is not exclusively incident to females, nor is it confined to the puerperal state. It is an inflammatory affection, but destitute of redness, one of the marks by which the other two species of inflammation are denoted; the name of phlegmasia alba, or white inflammation, will therefore sufficiently characterize this *variety* of it.

One of the most severe cases of white inflammation which has fallen under my observation, was in a lady who had suffered a miscarriage when about ten weeks advanced in her pregnancy; she was nearly exhausted by a violent flooding before assistance could be obtained, and when a little recruiting her strength, she was seized with this disorder. The tumefaction pervaded the whole of the right leg and foot from the knee downwards, was tense, painful and shining, and did not pit upon the pressure of the finger.

Her exhausted state precluded all idea of further evacuation, especially by bleeding; to the part, soothing and anodyne applications were directed; and on the subsidence of the tension, spirituous embrocations, aided by a roller to supply the loss of tone. Internally she took bark, and remedies of that class; together with such generous diet as the stomach could digest.

As the induration abated, a diffused deep-seated fluctuation was perceptible underneath, but as the integument did not any where shew a disposition to rupture, it was hoped that the fluid would be absorbed. One morning however a large quantity of a thin purulent matter was found in the bed, which it appeared had issued through a small circular opening in the calf of the leg. This I proposed to dilate, but she objected; and by elevating the limb and gently pressing out the matter at each dressing, with the assistance of a roller, the cavity was gradually obliterated. Nevertheless, many months elapsed before her health was re-established.

Besides the general swelling and induration, I have seen the phlegmasia alba more distinctly marked in the course of the trunks of the absorbents; and though for the most part an acute disease, I am very much mistaken if I have not seen it in a chronic form, and in an upper extremity. The last example which has occurred to me of an acute case, was in a man whose right testicle I had extirpated for a cancer; after the operation, every thing went on favourably until the wound was on the point of healing,

 when when he was attacked with a tensive tumefaction of the whole extremity on the same side, extending from the hip downwards. The disease yielded in this instance to the usual treatment.

The local characteristics of an external phlegmon are a circumscribed swelling, accompanied with redness, tension, and a throbbing pain; the redness and swelling are diffused in an erysipelas, and there is also a sense of ardent pain in the part: these two species of inflammation are however frequently combined, and as one or the other predominates, it has received the compound appellation of erysipelas-phlegmon, or phlegmon-erysipelas. One termination of phlegmon is by suppuration; the termination of erysipelas is by resolution or gangrene. In the erysipelas-phlegmon, or phlegmon-erysipelas, suppuration or gangrene, or both, may take place, according to the violence of each relatively, or their still greater virulence when combined. In conducting the treatment of this complicated form of disease, as the consequence most to be dreaded, our earliest endeavours will be directed to obviate gangrene by the use of the Peruvian bark, joining with it such correctives, as will enable the stomach to bear it in large and frequent doses, without load or oppression. If, on the contrary, the phlegmonous action should have taken the lead, leeching and other evacuations may be first employed; and then the bark will seldom fail in putting a stop to the erysipelatous affection, and curing the disease.

The local characteristics of phlegmasia alba, or white inflammation, agree in many respects with those of phlegmon and erysipelas; for, like erysipelas, it is diffused, and like phlegmon it is possessed of a considerable degree of tension. Pain and tumefaction are symptoms attendant on all the three species. In phlegmon, and in erysipelas, there is likewise a redness peculiar to each; but in the phlegmasia alba the surface is not discoloured, or in a few rare instances so little so, as not unaptly to receive this distinctive epithet. Like phlegmon too the duration of it will be shortened, or a cure effected by an antiphlogistic course of treatment.

The principal feature then in which the phlegmasia alba differs from the other two well known species of inflammation, is in the absence of redness upon the surface; but it would be just as philosophical to deny the existence of a muscle, because the fibres producing motion are white, as to say that an external disease possessing the other characteristics characteristics is not inflammatory, because an external redness did not form a constituent part of it. The tensive resistance in this malady is probably caused by the deposition of coagulable lymph. And it would appear from the case after a miscarriage above narrated, that a phlegmonous action of the vessels, which is denoted by the formation of matter, may exist in conjunction with the phlegmasia alba.

The manner in which the purulent fluid obtained a vent spontaneously in this case is deserving of notice. Instead of the thinning process, as in a common abscess, it was evacuated through a small circular aperture, similar to a hole made with a gimblet; and by an examination made with a probe, it had evidently lain upon the fascia covering the muscles below the thickened integument. Thus fortified against our researches the existence of pus cannot well be detected, or it is probable that suppuration is not an unfrequent occurrence.

From what is here stated, and other more minute points of similitude, I believe this disease to be a *variety* of inflammation, of which other unknown varieties may still exist; and each according to the state of the system at the time of the attack, the reigning diseases by which it may be modified, as well as the symptoms foregoing and attendant, will demand an appropriate treatment.

---

In the Number for December, 1805, p. 491, is a communication from Mr. Lodge Hall, entitled, 'An Attempt to improve the Screw Tourniquet;' and in a note he further observes, that 'The merit of this part of the improvement (or alteration) belongs to the ingenious artist who made the instrument from which the drawing (where it may be seen) was taken, Mr. Byrom, North Main Street, Cork."

The part of the tourniquet here referred to, is a transverse screw, the point of which is made to pass through a female screw in the upper plate, and rest in the groove of the perpendicular screw, and in principle and detail is nearly correspondent to the tourniquet which is engraved and described in the eighth volume of Medical Facts and Observations, of which Dr. Foart Simmons was the editor. I have been induced to notice this coincidence of sentiment, because my own tourniquet is, I think, free from an objection that will lie against the one which Mr. Hall has recommended.

In his tourniquet, the transverse screw has the head of a common screw, and consequently will require a driver to screw and unscrew it. On the contrary, in my own tourniquet the outer extremity of the transverse screw is made to project, and is winged, to enable the operator, or his assistant, to screw or unscrew it with the hand only.

But I trust that Mr. Hall will do me the favour to refer to the eighth volume of Medical Facts and Observations, for a more particular explanation than this.

## Figures and Tables

**Fig. 6 Fig. l Fig. 2 Fig. 3 Fig. 4 Fig. 5 Fig. 7. f1:**